# Commentary: Evaluation of the mechanical properties and clinical application of nickel–titanium shape memory alloy anal fistula clip

**DOI:** 10.3389/fsurg.2024.1367497

**Published:** 2024-03-27

**Authors:** Peter C. Ambe

**Affiliations:** ^1^ Department of Surgery, Vinenz-Pallotti-Hospital Bensberg, Bergisch Gladbach, Germany; ^2^ Department of Surgery, Witten/Herdecke University, Witten, Germany

**Keywords:** fistula-*in-ano*, anal fistula, OTSC (over-the-scope clip), fistula surgery, anorectal fistula

A Commentary on Evaluation of the mechanical properties and clinical application of nickel–titanium shape memory alloy anal fistula clip By Deng H, Li M, Fang X, Zhang J, Wang J, Tang K, Tang R, Jia R, Han Y, Shi Y, Dong Y (2023). Front. Surg. 10:1235666. doi: 10.3389/fsurg.2023.1235666

## Introduction

Anal fistula is a rather common condition in coloproctology and its management remains a challenge. Achieving healing without continence disturbance represents two relevant outcome measures from the patients’ perspective. These patient′s’ expectations are not always easy to meet, especially in cases with complex fistulae. Multiple fistula tracts, high trans-sphincteric fistula involving more than one-third of the sphincter apparatus as well as perineal fistula in Crohńs disease are well-known examples of complex fistulae. Low healing rates associated with high failure and/or recurrence rates are unfortunately common for such complex fistulae. Also, the risk of continence impairment following surgical management of such complex fistulae is high in comparison with cases with simple fistulae. Maintaining the integrity of the sphincter apparatus during surgical management of fistula-*in-ano* represents the most important aspect in preventing post-operative continence disturbance. This goal can be reached by choosing sphincter-preserving surgical options.

Fistula closure with a clip has been used as a sphincter-preserving technique over the past few decades. The use of a nitinol clip to manage anal fistula was first investigated by Prosst et al. in 2012, reporting a 90% healing rate with the Over-The-Scope Clips (OTSC) in an animal study (1). Prosst and Ehni in the same year published a clinical case reporting on the successful fistula closure using an OTSC (2). A randomized controlled pilot trial by Mascagni et al. comparing 15 patients managed with OTSC vs. 15 managed with fistulectomy and primary sphincter repair with low trans-sphincteric fistula showed a 93.3% healing rate for OTSC with reduced length of hospital stay and need for pain medication (3). Thus, the role of OTSC in the management of anorectal fistula was established,

Over the years, many series have been published with success rates varying between 20% and 80% (4, 5). The current literature on OTSC for the management of anorectal fistula is not only limited by the retrospective nature of the studies but very much also by the small sample sizes. More so, there is a high degree of heterogeneity in published studies with regard to follow-up and outcome measures. This probably explains why there seems to be no meta-analysis and systematic analysis on this intervention so far. Persistent post-operative pain requiring clip removal, clip migration, persistent discharge, and abscess formation have been reported as complications associated with OTSC surgery (4, 6). Although some of these morbidities may partly be associated with specific patient and disease characteristics (7), the safety of clipping anal fistulae remains a topic of debate. It is therefore not surprising that no recommendation could be made for the use of OTSC in the management of fistula-*in-ano* in the recently published European guidelines for the management of cryptoglandular fistula (8).

In their recently published study, Deng et al. evaluated the mechanical properties and clinical application of a nickel–titanium memory alloy anal fistula clip for the closure of anal fistula in 31 patients (9). The study population included mostly cases with complex fistula; high fistula, multiple tracts, and perianal Crohn’s. The outcomes of this experimental group were compared with those of a control group of 31 patients undergoing conventional fistula closure. No significant difference was seen between the study and the control group with regard to post-operative pain on the visual pain scale (3.59 vs. 3.52), healing rate (87.1% vs. 89.2%), and Wexner incontinence score (3.09 vs. 4.25). Pre-operative work-up and follow-up including endoanal ultrasound and magnetic resonance imaging (MRI) are in accordance with international standards. These results are better than what has been reported so far for fistula clips. Analyses of the cost-effectiveness compared with OTSC, the short follow-up of just about 6 months, missing information on clip-associated morbidity, and possible contraindications represent some limitations in this study.

## Discussion

The recently published European Society of ColoProctology (ESCP) guidelines for the diagnosis and management of cryptoglandular fistula could not spell out a definite recommendation on the use of OTSC in the closure of anal fistula based on the available evidence and experts’ opinion. This more or less “no recommendation” may be interpreted as a sign of caution regarding this intervention, at least for practitioners in Europe.

An interesting aspect of this study by Deng et al. may be the physical and chemical composition of the clip. While the OTSC used in the Western world is nitinol-based, that used in this study was a nickel–titanium alloy. Therefore, may be the composition of the clip is the game changer. As Aristotle once said: “what we have to learn to do, we learn by doing.” The results reported in this study from China should not be neglected by practitioners in the Western world, especially with respect to the “no recommendation” in the newly published European guidelines. The scientific community has the obligation to constantly explore and update the available evidence in their area of expertise. At times we just must learn by doing and the future will tell.
